# Embodiment and the origin of interval timing: kinematic and electromyographic data

**DOI:** 10.1007/s00221-016-4842-y

**Published:** 2016-12-09

**Authors:** Caspar Addyman, Sinead Rocha, Lilian Fautrelle, Robert M. French, Elizabeth Thomas, Denis Mareschal

**Affiliations:** 1 Department of Psychology, Goldsmiths, University of London, New Cross, London, SE14 6NW UK; 2 Centre for Brain and Cognitive Development, Department of Psychological Sciences, Birkbeck University of London, London, WC1E 7HX UK; 3 Unité de Formation et de Recherche en Sciences et Techniques des Activités Physiques et Sportives, Université Paris Ouest, Nanterre La Défense, Nanterre, France; 4 UMR 5022, Laboratoire d’Etude de l’Apprentissage et du Développement, Centre National de la Recherche Scientifique (CNRS), 21065 Dijon, France; 5 Unité de Formation et de Recherche en Sciences et Techniques des Activités Physiques et Sportives, Institut National de la Santé et de la Recherche Médicale (INSERM), U1093, Cognition, Action et Plasticité Sensori Motrice, Université de Bourgogne, Campus Universitaire, 21078 Dijon, France

**Keywords:** Interval timing, Infants, Electromyography, Embodiment, Open Data, Open Materials

## Abstract

Recent evidence suggests that interval timing (the judgment of durations lasting from approximately 500 ms. to a few minutes) is closely coupled to the action control system. We used surface electromyography (EMG) and motion capture technology to explore the emergence of this coupling in 4-, 6-, and 8-month-olds. We engaged infants in an active and socially relevant arm-raising task with seven cycles and response period. In one condition, cycles were slow (every 4 s); in another, they were fast (every 2 s). In the slow condition, we found evidence of time-locked sub-threshold EMG activity even in the absence of any observed overt motor responses at all three ages. This study shows that EMGs can be a more sensitive measure of interval timing in early development than overt behavior.

## Introduction

Interval timing concerns our ability to judge the length of events taking from a few seconds up to a few minutes (Buhusi and Meck 2005; Grondin 2008; Zakay and Block 1997). It is different from precision timing (occurring on a scale of less than 500 ms), which is involved in online motor control, and from long-term time perception (occurring on a scale of hours) involved in culturally specific notions of time. Interval timing or the “sense” of time passing is essential in structuring our interactions with the physical and social worlds. It is an ability that we share with many other species, and which appears to be present from early in human development. Indeed, over the last 15 years, substantial evidence using heart-rate measures (Colombo and Richman 2002), ERP measures (Brannon et al. 2004, 2008), looking time measures (Brannon et al. 2007), and eye-tracking measures (Addyman et al. 2014) has established that infants as young as 4 months old are sensitive to the unexpected interruption of a temporally regular event on the scale of several seconds.

But where does this ability come from? Our answer to this question starts from the view that action structures the sensory world. This is a classic developmental view that resonates with both the work of Piaget (e.g., 1952, 1957) and Eleanor Gibson (1969), as well as in more contemporary embodied approaches to perceptual and cognitive development (e.g., Thelen and Smith 1996; Goldfield 1995). Addyman et al. (2011; French et al. 2014) argue that the ubiquitous repetitive action cycles observed in young infants (e.g., Thelen 1979, 1981) can help calibrate temporal durations on the scale of interval timing.

There is, in fact, substantial evidence from studies with children with attention deficit and hyperactivity disorder (ADHD) and with adults that motor actions and temporal perception can mutually influence one another (see Kranjec and Chatterje 2010 for a full review). Firstly, conscious actions can alter adults’ perception of time (Gavazzi et al. 2013). For example, Haggard et al. (2002) showed that voluntary initiation of a button press shortened the time to the perceived onset of a resulting stimulus. This effect was reversed when movement was involuntarily induced with transcranial magnetic stimulation. Conversely, temporal awareness can affect the motor system. For example, Thomaschke and Dreisbach (2013) found that a predictable temporal pattern could improve the accuracy of motor responses but did not improve the accuracy of temporal judgments. Finally, recent work (e.g., Fautrelle et al. 2015; Carlini and French 2014) has revealed that, in adults, regular repeated motor activity improves interval timing accuracy at novel time scales. Participants’ motor responses to a regular target were improved by motor training or imagined motor training with a (different) regular target but not by mere observation or practice with an irregular target. Taken together, these studies suggest that, at least in adults, there is a close coupling between repeated regular movements and subsequent temporal judgment accuracy. Finally, children with ADHD show deficits in both interval timing and in motor coordination tasks, perhaps because both time perception and motor coordination depend on a right hemispheric fronto–striato–cerebellar network (Smith et al. 2003).

Studying the emergence of this coupling in infants is difficult because of their limited motor control. Thus, in the current article, we use surface electromyography (EMG) as an implicit measure of temporal anticipation and motor activity in young infants. Surface EMG recordings are noninvasive measures that allow the study of muscular activity through the electrical signal it emits. From the point of view of motor control, surface EMGs provide a window onto the neural commands used by the central nervous system to pilot the body. Importantly, EMG analyses allow us to measure anticipatory muscle activity and anticipatory postural adjustments (Bouisset and Zattara 1987; Hadders-Algra 2005; Thelen and Smith 1998; Witherington et al. 2002). These are essential for effective motor control because of the electromechanical delay in sending a command and realizing that command in the physical body (Schenau et al. 1995). In our case, EMG recordings and analyses will enable us to identify muscle activations synchronized with or anticipating rhythmic stimuli, whether they have observable kinematic consequences or not.

We focus on 4- to 8-month-olds because this is an age over which infants’ motor abilities change dramatically (Piek 2006). This is especially true for target reaching, object manipulations, and locomotion. It is, therefore, a critical age range with respect to the embodied timing hypothesis described above. We investigated interval-timing abilities directly in the context of a physical and socially interactive task. In our study, an experimenter raised an infant’s arms seven times at fixed intervals, but omitted to do so at the 8th interval, waiting to see whether the infant would initiate a time-sensitive movement during a pre-specified interval. This was repeated at two speeds, first on a slow 4-s cycle for four blocks; then, after a short break, on a fast 2-s cycle for four blocks. Responses were recorded using bipolar EMG and an infrared motion capture system. We hypothesized that, even if overt time-locked motor responses were not observed, evidence of temporal anticipation would be detected in the EMG signal.

## Method

### Participants

Fifty-seven infants took part in the 4-s condition comprising 20 four-month-olds (ten female; mean age = 129 days, range = 111 days to 142 days), 20 six-month-olds (14 female; mean age = 189 days, range = 157 days to 205 days), and 17 eight-month-olds (ten female; mean age = 243 days, range = 230 days to 267 days). Of these, 52 infants then took part in a faster 2-s condition including 16 four-month-olds (nine female; mean age = 128 days, range = 111 days to 141 days), 20 six-month-olds (14 female; mean age = 189 days, range = 157 days to 205 days), and 17 eight-month-olds (ten female; mean age = 243 days, range = 230 days to 267 days). Across both conditions, a total of 12 infants were excluded: eight infants because of fussiness or computer failure and four due to insufficient EMG data.

### Stimuli and procedure

**Fig. 1:**
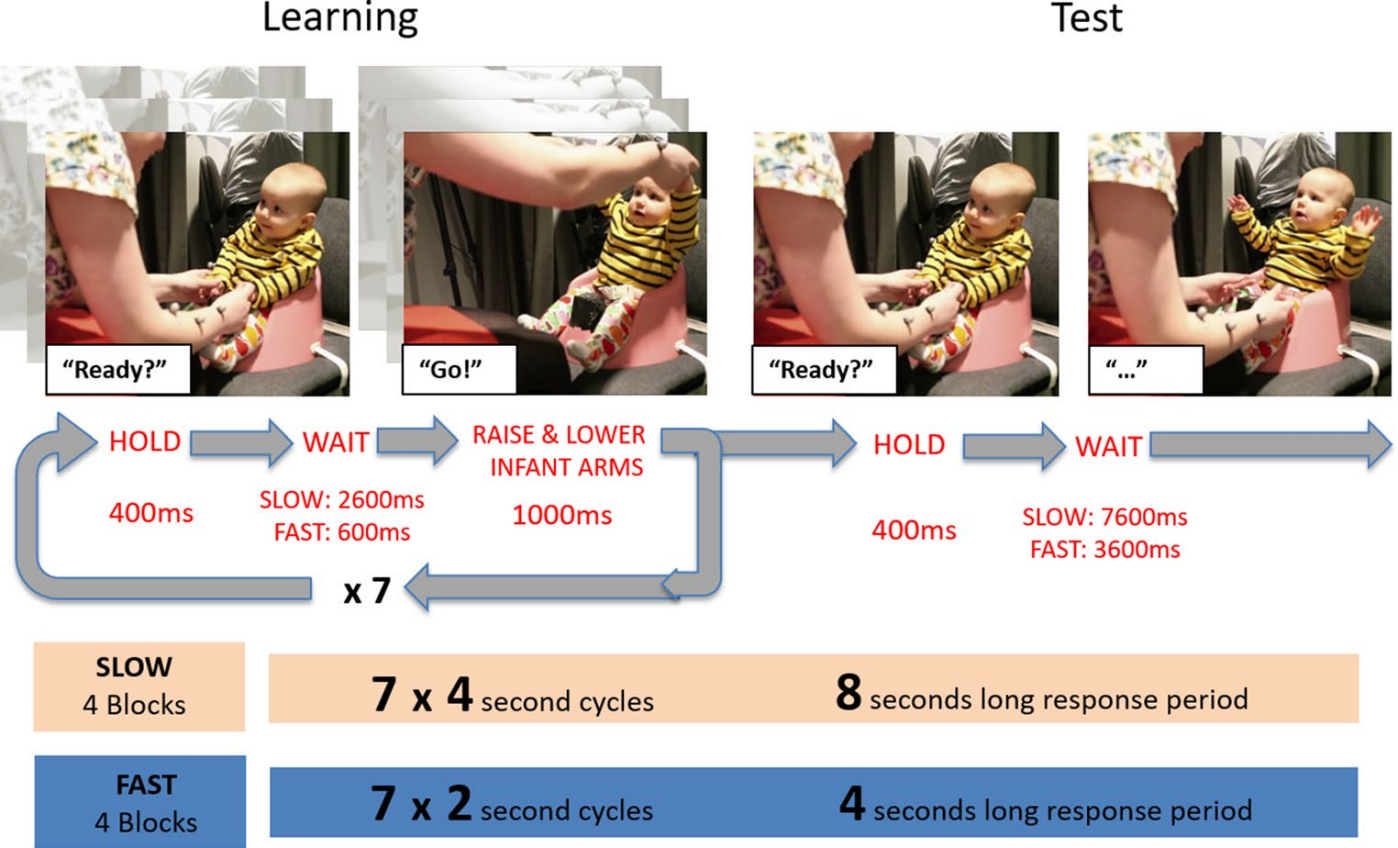
Schematic representation of hand-holding task. Infants completed four blocks of the slow version of the task (4-s cycles) had a break and then completed four blocks of the fast version (2-s cycles). In each block, an experimenter cued then lifted infants arms seven times in a *row* before finally cueing the infant and waiting for a response. The response period was twice the length of the learning cycle

Each condition (4- or 2-s intervals) consisted of four blocks of seven learning trials followed by one test trial (Fig. 1). During the learning trials, the experimenter, holding the infant’s hands in a relaxed position on the infant’s lap, gave the verbal cue “Ready?” (400 ms), paused (2600 ms or 600 ms), and then said “Go!,” while raising the infant’s arms to infant shoulder head height and lowering them back to the relaxed lap position (1000 ms). To ensure accurate timing, the experimenter had visual access to a monitor (behind and out of sight of the infant) displaying a colored ball and a numerical countdown of the trial duration. The beginning of the countdown prompted the experimenter’s verbal cue. At the end of the countdown, the ball moved from the bottom of the screen to the top of the screen and down again. The experimenter said “Go!” and lifted the infant’s arms in time with the ball on the screen. During the test trial, the experimenter used the verbal cue but remained in the relaxed lap position with her hands open for twice the length of a learning trial. In other words, she did not raise the infant’s arms during the test trial, but left the infant free to raise his/her arms.

Infants were seated either on a baby seat or on their carer’s lap opposite and within comfortable arm’s reach of the experimenter. Surface EMG was used to record the electrical activity of the infant and experimenter’s arm. Electrodes were placed on the infant’s upper left arm (*Bicep brachii*), chest (*Pectoralis major*), and left thigh (*Rectus femoris*), as well as on the experimenter’s right anterior deltoid (DE). Movements were simultaneously recorded through motion capture technology. Two spherical reflective markers (10 mm diameter) were attached to the infant’s sleeve over the left posterior forearm and connected as a free joint to three triangulated markers on the experimenter’s right anterior forearm. Thus, although the experimenter initiated a bilateral movement, only unilateral kinematic data were collected. In each block, the experimenter raised the infant’s hands seven times in a row (learning trials) on a fast (4 s) or slow (2 s) arm-lift cycle. On the eighth trial (test trial), the experimenter did nothing and waited for the infant’s spontaneous response.

### Apparatus

EMG data were collected using four bipolar pediatric surface electrodes (3 M monitoring electrodes with micropore tape and solid gel) and the Myon 320 wireless EMG system, at a sampling rate of 1000 Hz. Motion capture data were recorded using four infrared (IR) reference cameras (Bonita 10), inputting to a PC (Dell Precision T1600), at a sampling rate of 100 Hz. Synchronization of EMG and kinematic data was achieved using Vicon Nexus software (Version1.7.1). An in-house experimental control script written using Matlab^®^ R2009b generated the task timing signal for the experimenter to follow. This was displayed on a 17-inch screen out of view of the infant. This script also sent timing signals to the Vicon Nexus to mark the start of each cycle and each movement. This was achieved by sending transient on–off voltages directly to an unused EMG channel via an Arduino circuit driven by the MATLAB control script. All control scripts are included with the open dataset (Addyman et al. 2016b). Simultaneous video recording of the testing session was conducted using a webcam (Logitech HD 1080p) connected to the control PC.

### Data processing

Motion capture data were processed automatically by the Vicon Nexus software. The three-dimensional path coordinates of the infant arm markers were captured, reconstructed, and labeled by the Vicon software, following which, smoothing was performed using cross-validation splines (Woltring 1986). Trajectory data were filtered using a fourth-order Butterworth filter with zero lag and cut-off frequencies of 6 and 300 Hz. Relevant information concerning the infant arm movements was obtained from the marker *z* coordinates.

Raw EMG data were captured within the Vicon Nexus system providing synchrony with the motion capture data. However, all processing was performed in MATLAB using scripts written by the first author. Various methods have been used to interpret infant EMG signals (e.g., Van der Fits et al. 1999; Spencer and Thelen 2000; Nishida et al. 2006), with no established standard practice. We therefore used two independent analyses, one based on low-pass filtering and the other on a moving average, which are known to give similar shape and amplitude profiles (Winter 1990; Konrad 2005).

First, EMG data from the infant bicep were visually examined, and experimental blocks with noisy data were excluded. The remaining data (93%) were rectified and processed with a fourth-order Butterworth filter with zero lag and cut-off frequencies of 20 and 300 Hz using the MATLAB “filtfit” function. The data were then rectified a second time. These filtered data were then used in both analyses. For the continuous or analogue analysis, a low-pass fourth-order Butterworth filter was applied with zero lag and a 6 Hz cut-off. EMG amplitudes at selected points were then compared with each other. For the second binary method, we used a method identical to Van der Fits et al. (1999): For each block of data, a simple RMS moving average was calculated with a 200-ms window, which was then followed by calculating the first derivative of the smooth EMG trajectory. A moving baseline RMS for the preceding 3.4 s was also calculated. Activity was then coded as ON or OFF, where the moving average was, respectively, 1.4 times above or below the moving baseline (this is identical to the procedure reported in Van der Fits et al. 1999). From these data, we can then calculate the frequency of ON–OFF states or bursts in any given time window. All analysis scripts are included with the open dataset (Addyman et al. 2016b).

## Results

**Fig. 2:**
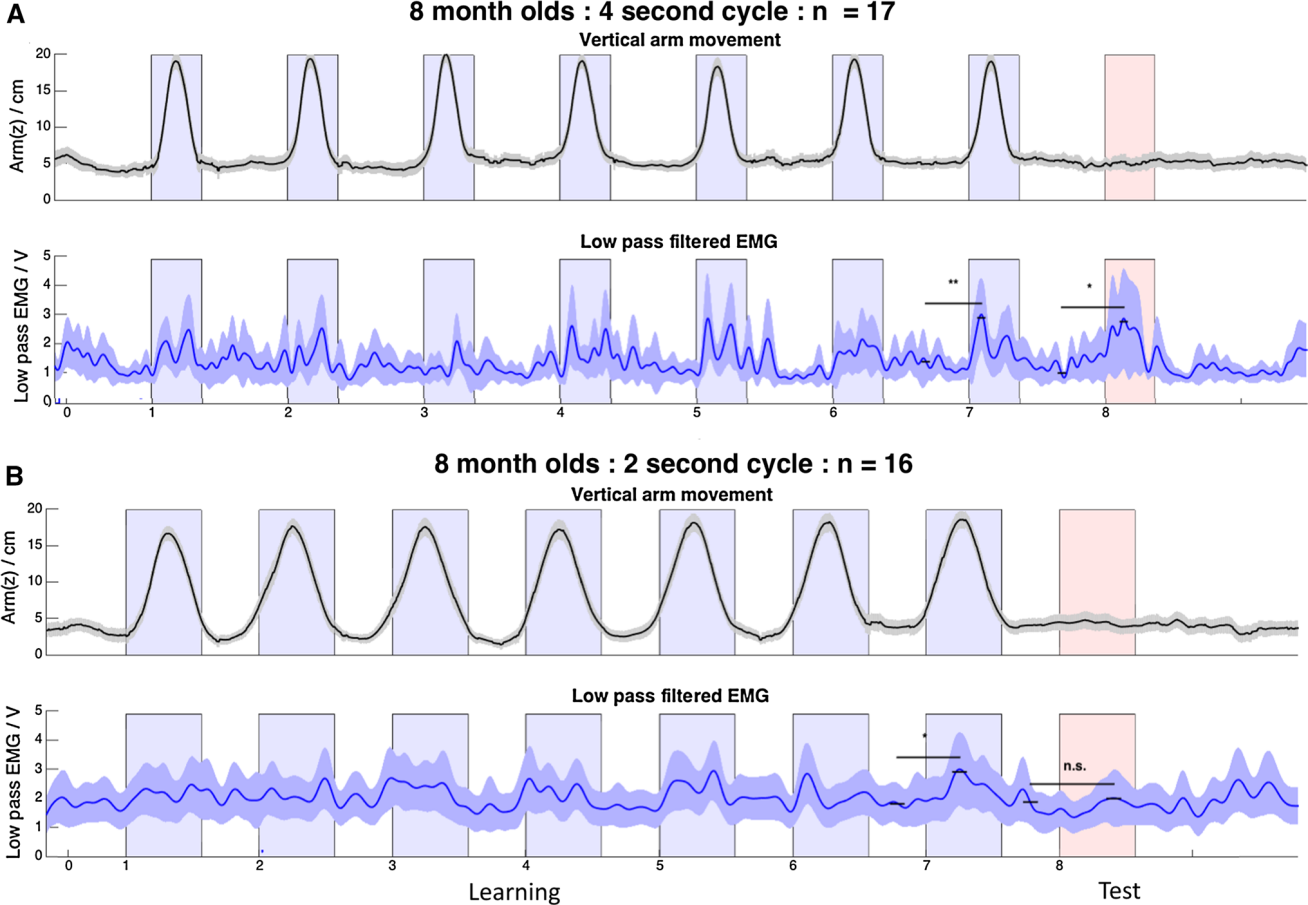
Average displacement in the *z* direction and average low-pass filtered EMG activity for 8-month-olds in the (**a**) 4-s, and (**b**) 2-s conditions. In each plot, the *dark line* represents the mean, and the *shaded area* is the 95% confidence interval. *Double asterisk* indicates *t* test significant at *p* < .005, *Single Asterisk* significant *p* < .05

Our hypothesis was that infants will learn to anticipate the hand rising during the first seven training episodes and so will show time-locked movement and associated muscle activity in the subsequent missing beat period. We were therefore specifically interested in the infants’ data in the missed-beat test periods (time period 8) and how this compares to the movement and activity in the experimenter-led training periods (time periods 1–7). Infants’ responses were measured by looking at the movement along the z-dimension of the marker on the infant’s wrist and by the timing of sustained bursts of bicep EMG activity during the test period. We first grouped the infants by age and by condition, then collapsed the data across the four trial blocks for each baby in each timing condition. Using the EMG timing signal from the MATLAB control script, we were able to synchronize the starts of all the blocks so that the data could be superimposed on each other. As is done with ERP data, we aligned the EMG data in this way and averaged across events to explore group performance. For the purposes of illustration, the data recorded from 8-month-olds are shown in Fig. 2. As shown by the movement deflections in the *z* direction, the infants participated fully in the learning trials, letting the experimenter raise and lower their hands at the appropriate times. However, there was no evidence of overt (i.e., visible from the kinematics) response in the critical test period at either cycle length.

### Continuous EMG data analysis

Although the kinematic data suggest that infants were not reacting in the test period (i.e., interval 8), previous research has shown that infant responses can be sub-threshold for actual movement when anticipating interaction with a social partner (e.g., Reddy et al. 2013) or when making postural adjustments in preparation for a reach action (van der Fits et al. 1999; Witherington et al. 2002; Thelen and Spencer 1998). The EMG recording from the infants’ left biceps allows us to examine this. The second row in Fig. 2a, b shows the average low-pass filtered EMG signal for the 8-month age group and interval length, averaged across the four test trials for each baby. In the 4-s condition, each raising of the arms (time points 1–7) is accompanied by an increase in EMG activation, while each lowering is accompanied by a decrease in EMG activity. This is also the case in the 8th (test) trial, even though no overt movement was observed. In the 2-s condition, the same pattern is found during the training phase, but not in the test trial.

Thus, it appears that infants in the slow 4-s condition show a time-locked motor response, albeit one that is sub-threshold and not accompanied by overt movement. To test this hypothesis statistically, we averaged the activations over a 200-ms interval situated in the middle of the down part of the cycle (trough value) and compared this to the groupwise highest activation in the response period (peak value) in the last learning trial (shaded blue) or in the test trial (shaded red). These values are displayed in top three rows in Table 1. A mixed analysis of variance was carried out with Age (4, 6, or 8 months) as a between-subject factor and Trial Type (learning vs test) and Cycle Point (trough, peak) as a within subject factors. This analysis revealed a main effect of Age, *F*(2, 54) = 3.69, *p* = .032, partial eta squared = .12, reflecting a lower mean voltages in the oldest age group. There were also main effects of Trial Type, *F*(1, 54) = 11.06, *p* = .002, partial eta squared = .17 and of Cycle Point *F*(1, 54) = 22.75, *p* < .001, partial eta squared = .30. These main effects were modulated by a significant Trial Type × Age interaction *F*(2, 54) = 3.21, *p* = .048, partial eta squared = .11 and a marginally significant Trial Type × Cycle Point interaction *F*(1, 54) = 3.95, *p* < .052, partial eta squared = .068. Responses were larger in the learning period as compared to test period, and the response during test trials differed with age. This interpretation was confirmed using post hoc *t* tests (see Table 1, top 3 rows). No other interaction was significant.

**Table 1:** Comparison of EMG activities in critical points in the experiment

Task timing	Age	*N*	Final learning trials				Test trial			
			Cycle 6 arms down	Cycle 7 arms up	*t* test	*p*	Cycle 7 arms down	Cycle 8 arms up	*t* test	*p*
SLOW 4 s	8 m	17	.045 ± .12	.095 ± .18	2.60	.006**	.032 ± .05	.091 ± .25	1.81	.036*
	6 m	20	.11 ± .17	.28 ± .41	4.56	.001***	.14 ± .25	.17 ± .30	.97	.17
	4 m	20	.16 ± .28	.32 ± .40	2.97	.002**	.10 ± .15	.17 ± .28	1.87	.032*
FAST 2 s	8 m	16	.11 ± .20	.18 ± .36	1.68	.049*	.12 ± .22	.12 ± .27	.25	.40
	6 m	20	.17 ± .28	.28 ± .39	2.92	.002**	.16 ± .21	.16 +.25	.02	.49
	4 m	16	.20 ± .32	.31 ± .38	2.46	.008**	.24 ± .37	.24 ± .38	.1	.54

The middle set of columns compares peak and troughs in final learning trial, while rightmost columns compare same points in test period

Columns show mean (±SD) activation, one-tailed repeated measures *t* tests, and *p* values. (* *p* < .05; ** *p* < .01; *** *p* < .001)

An identical (3 × 2 × 2) ANOVA was performed on the 2-s data. At this time, there was no main effect of Age, *F*(2, 52) = 1.70, *p* = .192, but there were main effects of Trial Type, *F*(1, 54) = 5.63, *p* = .021, partial eta squared = .10 and of Cycle Point *F*(1, 52) = 7.98, *p* = .007, partial eta squared = .13. These main effects were modulated by a significant Trial Type × Cycle Point interaction *F*(1, 52) = 8.64, *p* = .005, partial eta squared = .14. No other interactions were significant. In this condition, a consistent pattern of response was seen across all ages with a clear difference between peak and trough at the end of learning, but no differences with age of the response during the test trial. This interpretation was confirmed using post hoc *t* tests (see Table 1, bottom 3 rows).

### Binary EMG data analysis

We ran a similar analysis on the ON–OFF burst frequency data (Table 2). These are binary data, so we therefore adopted a nonparametric approach. First, we compared the average burst frequencies at the same time points identified in the first analysis using one-tailed Wilcoxon signed-rank tests. This analysis revealed a highly significant trough-to-peak difference for all age groups during the learning cycles. During the test phase, this trough-to-peak difference was significant for the 6- and 8-month-olds, and marginally significant for the 4-month-olds, in the 4-s condition. There were no significant differences at any age in the 2-s condition.

**Table 2:** Average number of ON bursts per infant per trial during middle of arms down and arms ups intervals during final learning trial and test trial

Trial timing	Age (m)	*N*	Final learning trials			Test trials		
			Cycle 6 arms down	Cycle 7 arms up	Sign test *p*	Cycle 7 arms down	Cycle 8 arms up	Sign test *p*
SLOW 4 s	8	17	.10	.34	.001***	.06	.15	.04*
	6	20	.06	.36	.001***	.10	.21	.01**
	4	20	.08	.30	.001**	.08	.13	.06
FAST 2 s	8	16	.09	.22	.01*	.12	.11	.64
	6	20	.10	.22	.01*	.10	.16	.12
	4	16	.09	.26	.002**	.14	.17	.38

Columns show mean number of bursts and *p* values from one-tailed Wilcoxon signed-rank tests (* *p* < .05; ** *p* < .01; *** *p* < .001)

## Discussion

This study investigated 4-, 6- and 8-month-olds’ responses to a regularly repeating socially driven motor interaction. An experimenter raised the infant’s arms seven times in a row at regular intervals. We then observed the infant’s response in a ‘missed-beat’ interval. Infants were tested on slow (4 s) and fast (2 s) cycles. We found no evidence of behavioral (i.e., overt) anticipation at any age or time scale, in that motion capture data showed the infants did not raise their arms in the test response period. However, low-pass filtered EMGs from infants’ left biceps revealed a significant time-locked signal in the 4-s condition for 4- and 8-month-olds. A similar time-locked response was observed in the burst frequencies of 6- and 8-month-olds, in the 4-s condition. No EMG effects were found at any age in the 2-s condition. Thus, across the two measures, we found evidence of time-locked EMG activity in all three age groups, with the most robust response (as evidenced in both measures) present in the older 8-month-old group only.

How do these findings relate to what is already known about infant timing ability? Although there has been no previous research on infant motor timing, our results are consistent with more general surprise-based measures of timing ability. These include evidence of physiological correlates of time discrimination detected in heart-rate variation at 4-month-olds (Colombo and Richman 2002), visual preference at 6-months (Brannon et al. 2007), and in event-related potentials (ERPs) in 10-month-olds (Brannon et al. 2008). The current results are also consistent with our previous work using an event-based paradigm in the visual domain (Addyman et al. 2014), which found evidence of interval timing from 4 months of age with no developmental trend.

The results from the faster time cycle stand in contrast to previous reports suggesting that infants as young as 4 months of age are sensitive to interruptions of temporal regularity (e.g., Addyman et al. 2014; Colombo and Richman 2002). There are two reasons for why this discrepancy might have appeared. First, the current task requires a volitional upper-body response from the infant rather than just anticipatory eye movements, reflexive pupil dilation, or heart-rate deceleration of previous studies. The physical response is more complex and requiring coordination of numerous postural muscles which develops slowly in the first 18 months (Van Der Fits et al.^1999^). The second reason is that this task involved a highly engaging—and consequently potentially distracting—social interaction that may have captured the infant’s attention and undermined their ability to demonstrate fully their accurate time-keeping abilities.

Investigating interval timing in infants is very difficult. Researchers must balance the need to expose infants to a stimulus that extends through time and which is repeated sufficiently often for the infant to learn the regularity, while simultaneously avoiding boredom and the loss of attention. The current research introduces a new paradigm to the study of timing of infants under one year of age, and crucially, demonstrates that even in the absence of an observed overt response, EMGs can carry time-locked information revealing the infant’s expectations about the onset of a stimulus occurring on the interval time scale. Thus, EMGs are a more sensitive measure of infant timing abilities than are overt behavioral responses; EMG methods reveal proficiency in the physical domain that these latter methods have not previously detected.

This is important because understanding the very early development of interval timing places strong constraints on the plausibility of timing models in older children and adults (See Addyman et al. 2016a, for a recent review). Our findings are consistent with the suggestion that embodied experience is essential to acquisition of cognitive skills (e.g., Kermoian and Campos 1988). We provide evidence for an emerging coupling between the motor system and interval timing. In this sense, interval timing is indeed embodied (cf. Kranjec and Chatterje 2010; French et al. 2014).

We believe that infant interval timing abilities are highly embodied in a “Gibsonian” or ecological sense. Namely, it is the infant’s physical interactions with the world that provides much of the structure to help develop their internal representation of time. Eleanor and James Gibson proposed that action and perception are tightly coupled to an environment that provides physical affordances (Gibson 1982; Gibson 1979). Although the term “affordance” is often used in relation to objects, the environment or ecological setting provide affordances too. This is sometimes also referred to as the “unity of perception” (Thelen 1995). Thus, we suggest that object manipulation and locomotion will both play an important role in the early development of time perception. When infants successfully reach for and manually explore objects in their peripersonal space, they will be afforded a much richer temporal experience than when they were just passive observers. Moving around affords even larger temporal changes in the world (the room looks very different from over here), and temporal judgments become more important (how long will it take me to get to that toy?).

## Conclusion

The current study introduces a new event-based paradigm for investigating infant time perception that allow for the first time the investigation of motor components of interval timing. Moreover, it demonstrates how EMGs can be more sensitive measures of infant interval timing abilities than overt observed behavior. Finally, these results are consistent with an embodied developmental model of interval timing.
